# Supplementary material for: A Heterogeneously Expressed Gene Family Modulates the Biofilm Architecture and Hypoxic Growth of Aspergillus fumigatus
Source: mBio. 2021 Feb 16;12(1):e03579-20. doi: 10.1128/mBio.03579-20 (PMC8545126; doi:10.1128/mBio.03579-20)
Supplement: FIG S1 [file mbio.03579-20-sf001.pdf]

A

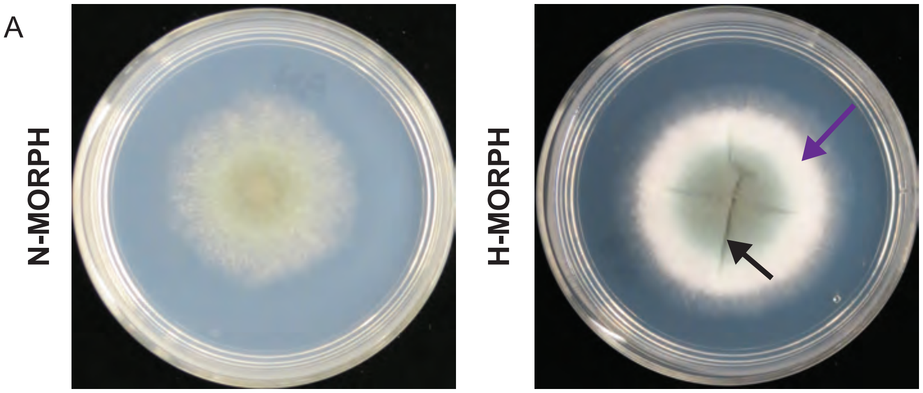

B

AF293 Chromosome 5:

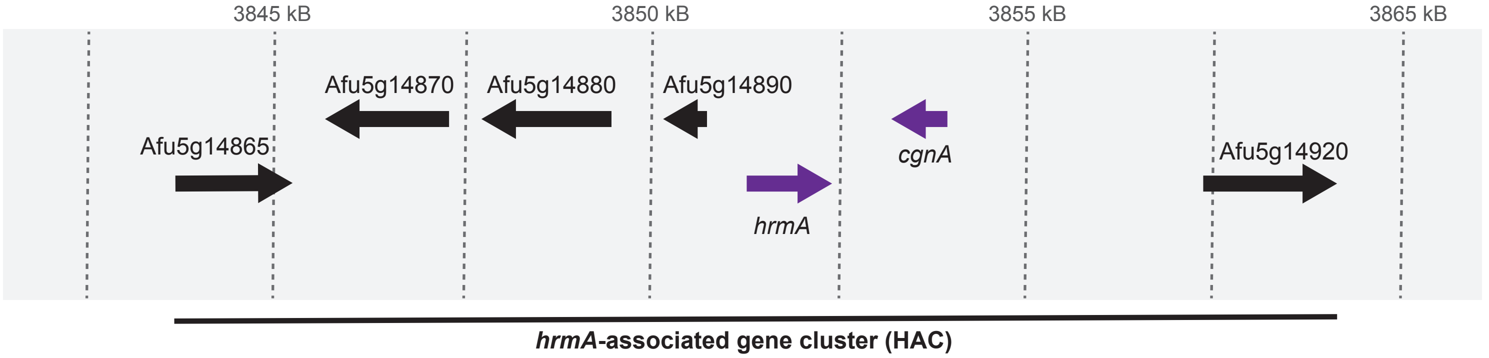

C

Strain	Colony Morphology	Biofilm Morphology	H/N	Adherence	*hrmA* allele	HAC expression
AF293	N-MORPH	Vertical	Baseline	Baseline	Wild Type	LOW
AF293 Δ*hrmA*	N-MORPH	Vertical	Baseline	------	n/a	LOW
AF293 Δ*cgnA*	N-MORPH	------	Baseline	Baseline	Wild Type	LOW
EVOL20 Δ*hrmA*	N-MORPH	Vertical	Slightly Increased	------	n/a	LOW
EVOL20 Δ*cgnA*	N-MORPH	Vertical	Baseline	Baseline	Evolved	HIGH
AF293 *cgnA*^{OE}	N-MORPH	------	Baseline	Baseline	Wild Type	------
EVOL20	H-MORPH	Horizontal	Increased	Reduced	Evolved	HIGH
AF293 *hrmA*^{OE}	H-MORPH	------	Increased	Reduced	Wild Type	HIGH
AF293 *hrmA*$^{R-EV}$	H-MORPH	Horizontal	Increased	Reduced	Evolved	HIGH
