# Supplementary figures and images for: A Heterogeneously Expressed Gene Family Modulates the Biofilm Architecture and Hypoxic Growth of Aspergillus fumigatus
Source: mBio. 2021 Feb 16;12(1):e03579-20. doi: 10.1128/mBio.03579-20 (PMC8545126; doi:10.1128/mBio.03579-20)

A

Representative Samples from Normoxia RNA-sequencing (Kowalski et al. 2019)

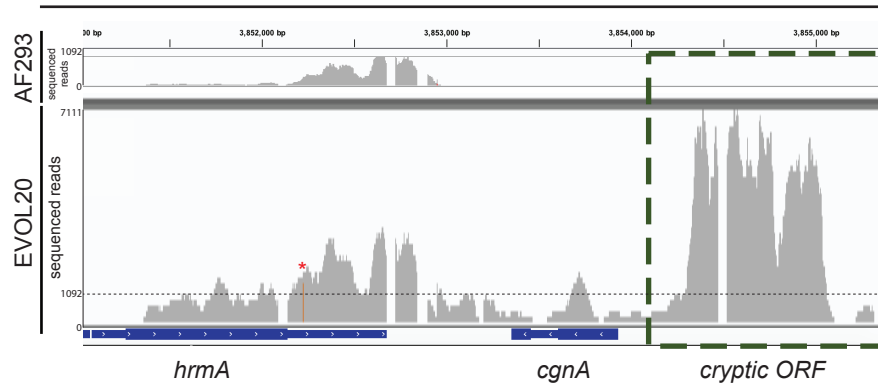

B

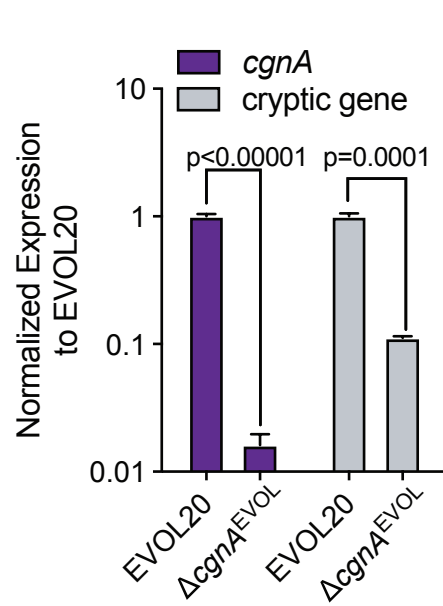

C

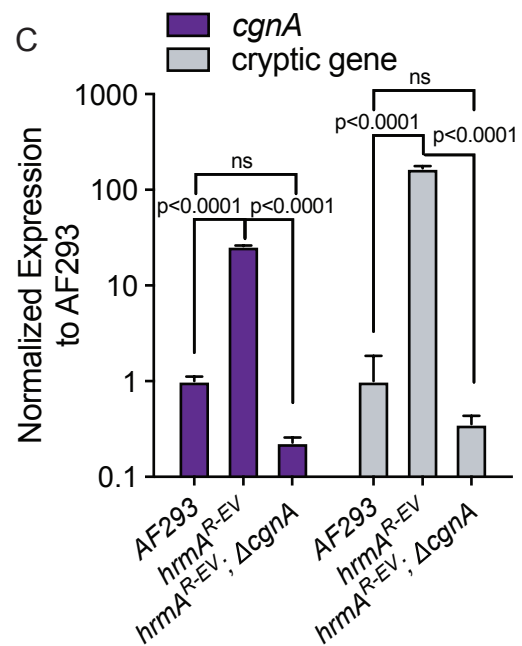

D

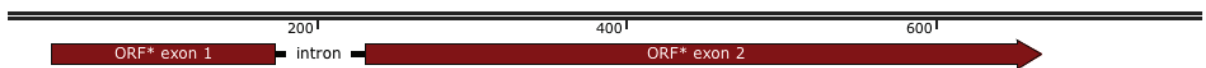

E

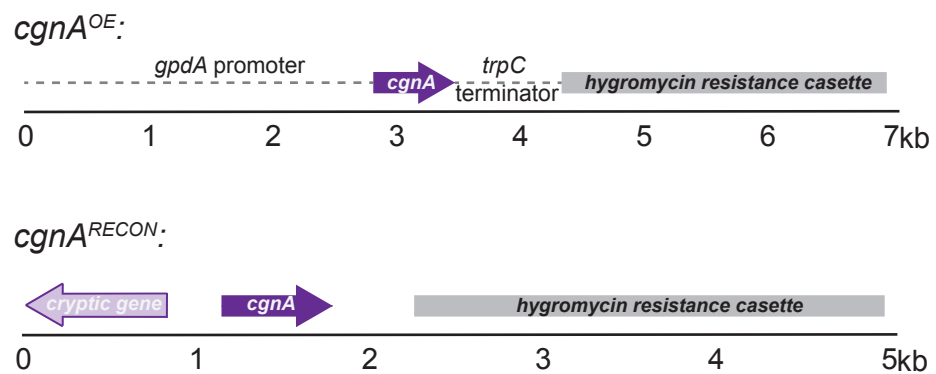

Supplement: FIG S2 [file mbio.03579-20-sf002.pdf]

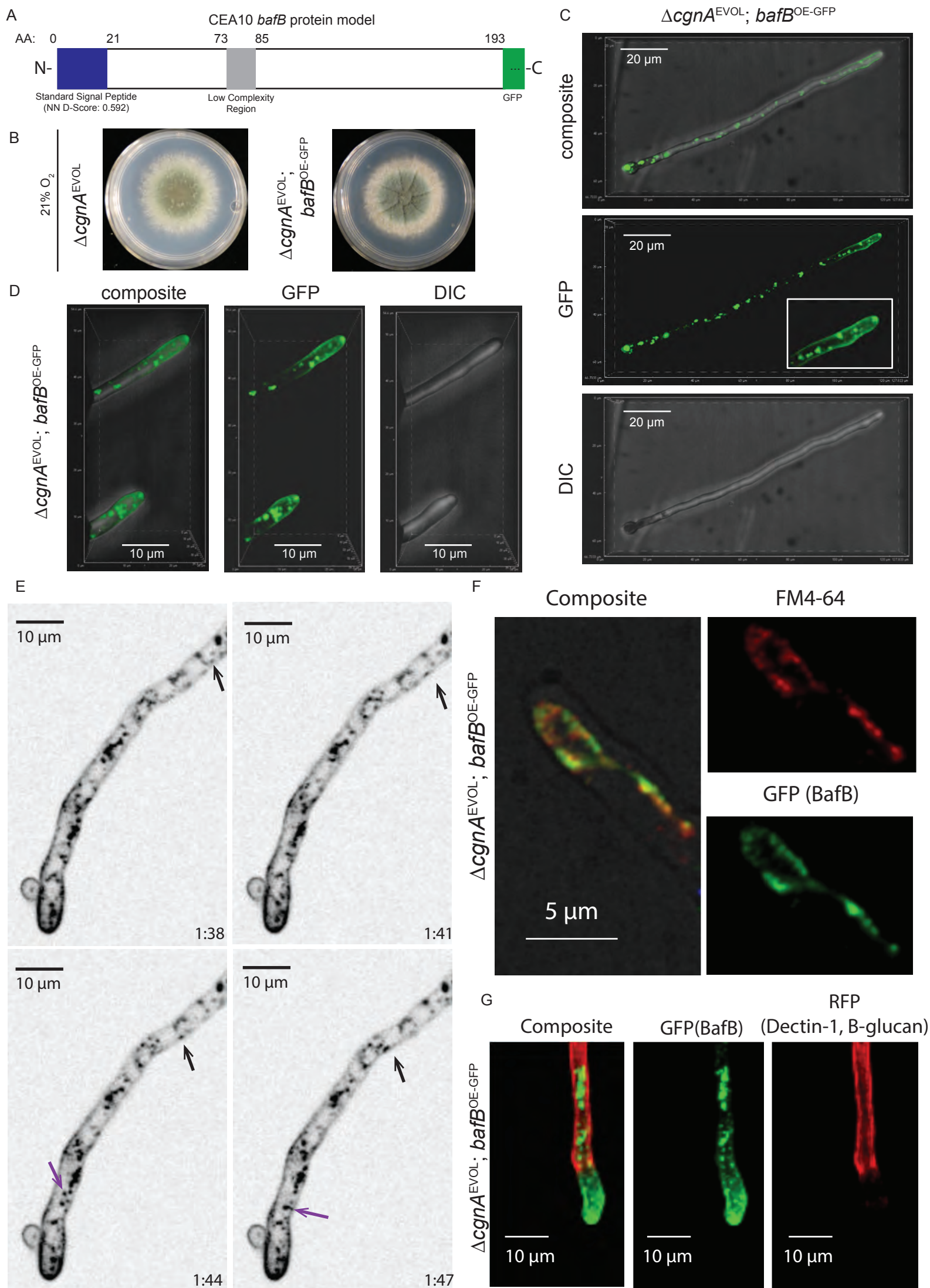

Supplement: FIG S5 [file mbio.03579-20-sf005.pdf]

A

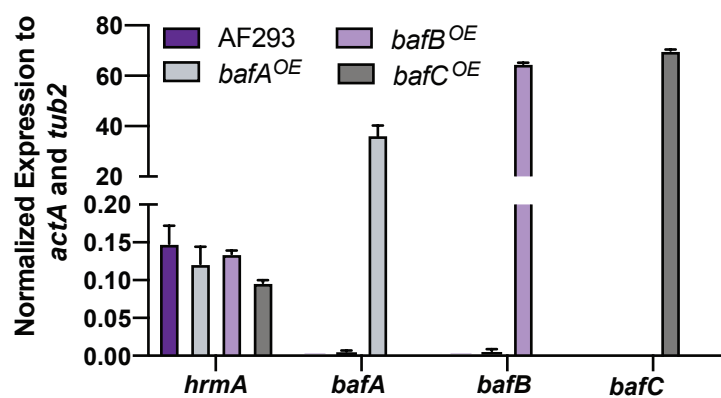

B

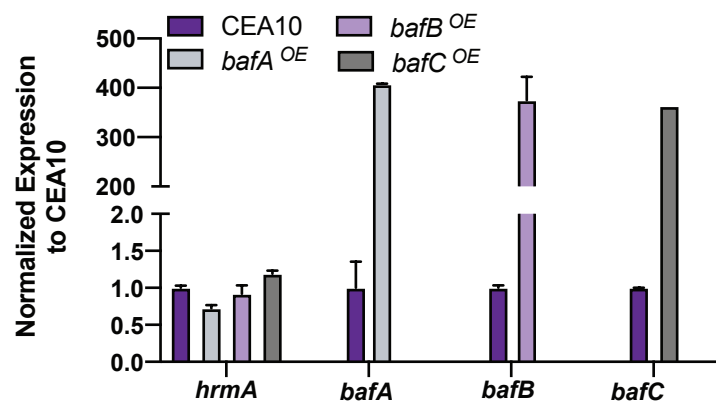

C

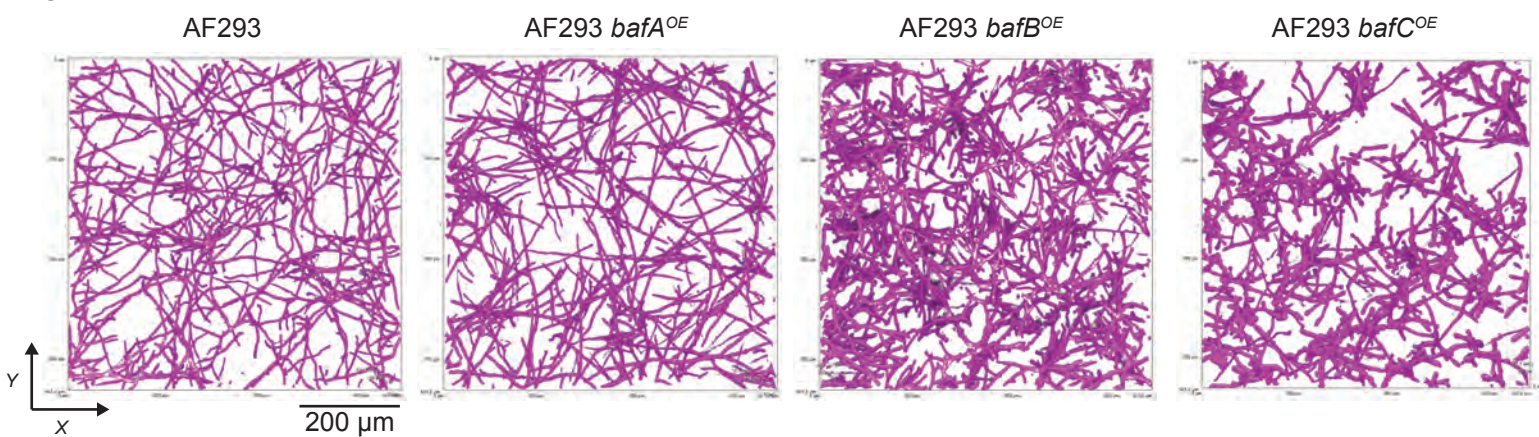

D

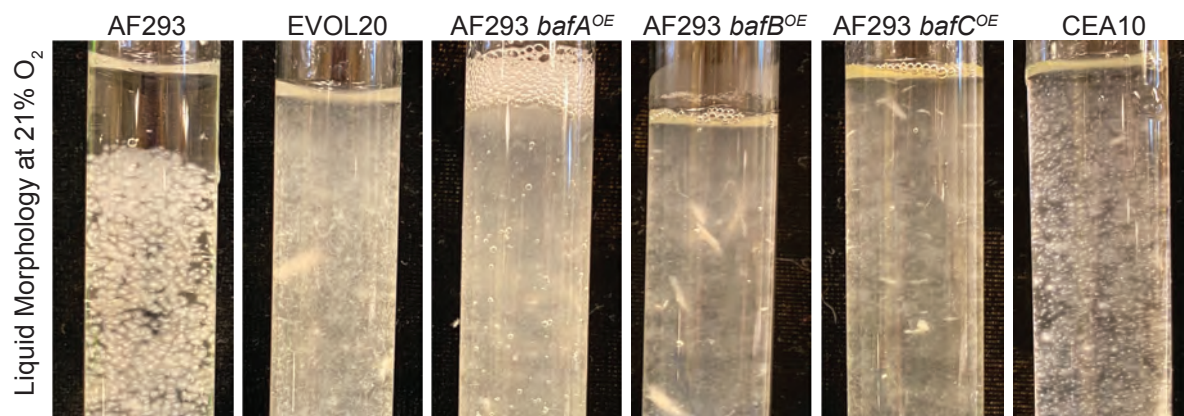

E

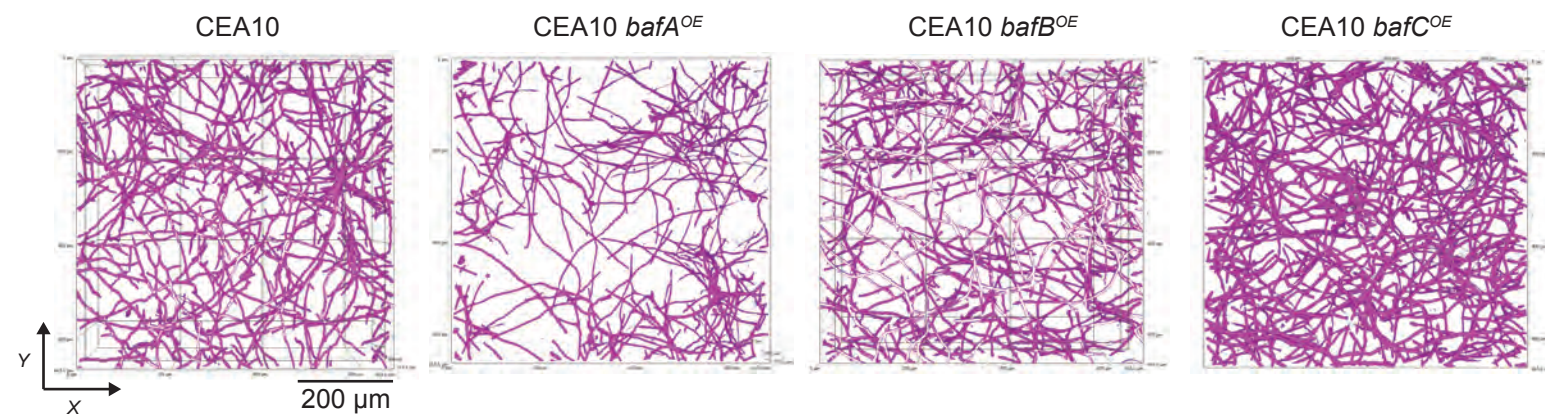

Supplement: FIG S6 [file mbio.03579-20-sf006.pdf]
