# Supplementary material for: A Heterogeneously Expressed Gene Family Modulates the Biofilm Architecture and Hypoxic Growth of Aspergillus fumigatus
Source: mBio. 2021 Feb 16;12(1):e03579-20. doi: 10.1128/mBio.03579-20 (PMC8545126; doi:10.1128/mBio.03579-20)
Supplement: TABLE S1 [file mbio.03579-20-st001.pdf]

Strain ID	Strain Genotype	Origination
AF293	Wild-type	Nierman et al 2005
CEA10	Wild-type	Girardin et al 1993
EVOL20	Hypoxia passaged AF293	Kowalski et al 2016
hrmA$^{R\text{-}EV}$	AF293.1 Δ*hrmA; pyrG*⁺; *hrmA*^{EV}; *ptrA*⁺	Kowalski et al. 2019
hrmA$^{R\text{-}EV}$*;* Δ*cgnA*	AF293.1 Δ*hrmA; pyrG*⁺; *hrmA*^{EV}; ptrA⁺; Δ*cgnA*; *hygB*⁺	Kowalski et al. 2019
Δ*cgnA*^{EVOL}	EVOL20 Δ*cgnA*; *ptrA*⁺	Kowalski et al. 2019
Δ*hrmA*^{EVOL}	EVOL20 Δ*hrmA*; *ptrA*⁺	Kowalski et al. 2019
Δ*cgnA*^{EVOL}; *cgnA*^{RECON}	EVOL20 Δ*cgnA*; ptrA⁺; *cgnA*; *hygB*⁺	This study
Δ*cgnA*^{EVOL}; *cgnA*^{OE}	EVOL20 Δ*cgnA*; ptrA⁺; gpdA-*cgnA-trpC*; *hygB*⁺	This study
Δ*hrmA*^{EVOL}; *bafB*^{OE}	EVOL20 Δ*hrmA*; *ptrA*⁺; *gpdA-bafB-trpC; hygB*⁺	This study
Δ*cgnA*^{EVOL}*; bafB*^{OE}	EVOL20 Δ*cgnA*; *ptrA*⁺; *gpdA-bafB-trpC; hygB*⁺	This study
AF293 *bafA*^{OE}	*gpdA-bafA-trpC*; *ptrA*⁺	This study
AF293 *bafB*^{OE}	*gpdA-bafB-trpC*; *hygB*⁺	This study
AF293 *bafC*^{OE}	*gpdA-bafC-trpC*; *hygB*⁺	This study
CEA10 *bafA*^{OE}	*gpdA-bafA-trpC*; *ptrA*⁺	This study
CEA10 *bafB*^{OE}	*gpdA-bafB-trpC*; *hygB*⁺	This study
CEA10 *bafC*^{OE}	*gpdA-bafC-trpC*; *hygB*⁺	This study
An A1144 (FGSC A1144)	Wild-type	Fungal Genomics Stock Center
An *AfbafA*^{OE}	*gpdA-AfbafA-trpC*; *ptrA*⁺	This study
Δ*cgnA*^{EVOL}; *bafB*$^{OE\text{-}GFP}$	EVOL20 Δ*cgnA*; *ptrA*⁺; *gpdA-bafB-GFP-trpC*; *hygB*⁺	This study
