# Supplementary material for: A Heterogeneously Expressed Gene Family Modulates the Biofilm Architecture and Hypoxic Growth of Aspergillus fumigatus
Source: mBio. 2021 Feb 16;12(1):e03579-20. doi: 10.1128/mBio.03579-20 (PMC8545126; doi:10.1128/mBio.03579-20)
Supplement: FIG S3 [file mbio.03579-20-sf003.pdf]

A

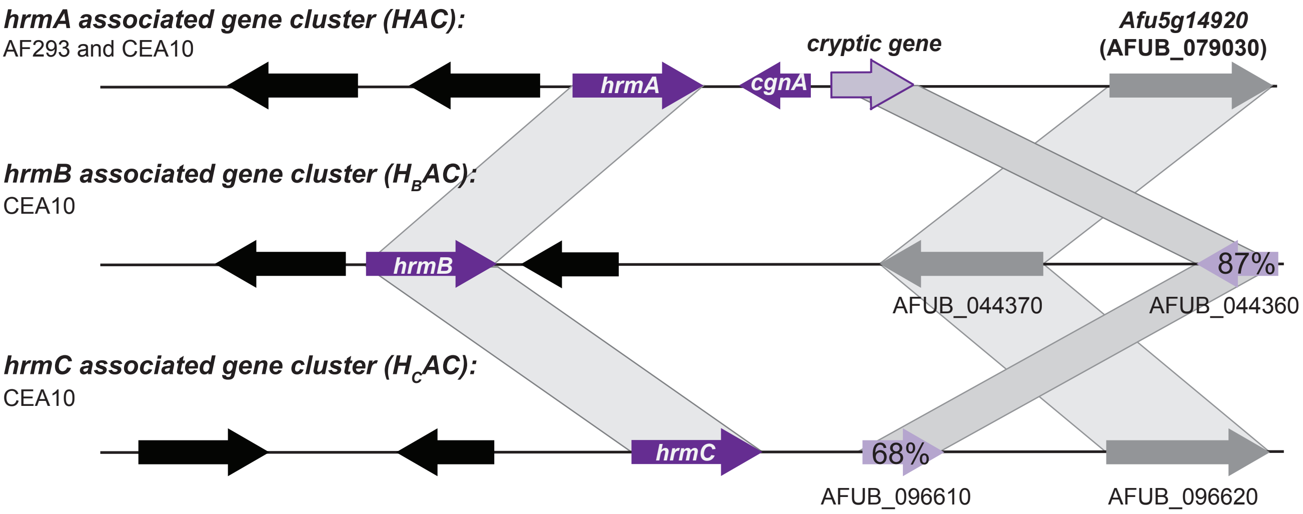

B

```
ABUB_044360      MVWYRAILVCMPWWL--MGRNSTNEGKRSEGERAPMIDKVPTFEEMTITTSAKYVNGEKI   58
Predicted        MAWYRALLPCIPLWRKVLGRNSTDEDGRSEDDLTSLSDKMPTFEET--TTSAKYINGEKI   58
                 *.****:* *:* *   :*****:*. ***.: : : **:*****   ******:*****

ABUB_044360      MEHTVVETKQIDNRGDTSVSNNDSNSTAETRHSGLSSVSHSDQSKVVEDANALEKPELFA   118
Predicted        MEHTVVETKHIDERGDTSVSNGDSNSTAVTRHSGLSSVSLSDQSTIVEDANALEEPELFA   118
                 *********:**:********.****** ********** ****.:********:*****

ABUB_044360      VHSPYVDASTGKQMLRLYYELPVSLDDLEITGLESRIPESDDDSIEACFCYRGEKFWLHV   178
Predicted        VHSPYVDDSTGEQMVRLYYELPVSLDDLEIIGLESRIPESDDDSIEARFRYRGEDFWLPV   178
                 ******* ***:**:*************** **************** * ****.*** *

ABUB_044360      PYSYAKARMVLMGVY       193
Predicted        RYSYAKARMVLTGVC       193
                  ********** **
```

C

```
Predicted        MAWYRALLPCI-PLWRKVLGRNSTDEDGRSEDDLTSLSDKMPTFEETT--TSAKYINGEK   57
AFUB_096610      MAWYEVFEQWVYWCWQRIWPFDDSRRDGRNEDDLTSLTDKMPVFEDKIINTSVRYVNGEI   60
                 ****..:   :   *:::   :.: .***.*******:****.**:.   **.:*:***

Predicted        IMEHTVVETKHIDERGDTSVSNGDSNSTAVTRHSGLSSVSLSDQSTIVED--ANALEEPE   115
AFUB_096610      AA--YVVQTQYLDTQEVSSARDSYWKSVADIKPGDFCSHSISDQSTIVEENEAKALEGPE   118
                      **:*:::* :  :*. :.  :*.*  : ..:.* *:********:  *:*** **

Predicted        LFAVHSPYVDDSTGEQMVRLYYELPVSLDDLEIIGLESRIPESDDDSIEARFRYRGEDFW   175
AFUB_096610      PFAVRPSYIG-STGKRTVDFFYKVSLPLDDLEMRDKESRVPESSEDLIEALFHYQGADIW   177
                  ***:  *:. ***:: * ::*:: : *****: . ***:***.:* *** *:*:* *:*

Predicted        LPVRYSYAKARMVLTGVC-    193
AFUB_096610      VYVPYSYANARMVSGGPTE    196
                 : * ****:****  *
```
