# Supplementary material for: A Heterogeneously Expressed Gene Family Modulates the Biofilm Architecture and Hypoxic Growth of Aspergillus fumigatus
Source: mBio. 2021 Feb 16;12(1):e03579-20. doi: 10.1128/mBio.03579-20 (PMC8545126; doi:10.1128/mBio.03579-20)
Supplement: FIG S4 [file mbio.03579-20-sf004.pdf]

A

H_BAC gene	AF293 Chromosome 3 Genome Location (bp)	Percent nt identity
hrmB	1023704 - 1025056	93%
bafB	1051538 - 1050966	89%
AFUB_044370	1049272 - 1047590	92%
AFUB_044380	1042710 - 1041989	95%

B

AF293 ‘*hrmB*’ Chr. 3 Region Translated

MARSNYLRIFRVLEEINQRHTIPNIYYEALEDCLVITFTQPSGPLKQSKTCYLKKEYKLQ
YKNTNTHRVYVEILEDYPQLFIPFILATPPKSCETFKLGEFREKHDLSAVKVDLRPNIQQ
TLDNITNQKGFNQNR*Y*YLICMLFPLGRKPATTAET*NCWAYRAAYLNTVHTIFSK*IY
SAMEVSLTAHSPECQAPQTTSCVEMKLPKQNYQDAIVLLELSLLIDIIKILFPSANE*II
CSLSPQSGRGLEPLTQITEPGHHNS*SE*STISQSKGYIL*GASISAILSIFSPCI*GAI
EKS*LRK*EKDNLSEETTDCILIKIHLRRPHCSTCRV*IRFINDSYFRRKTVHLSLSLSA
PNLLALLILEFNIFVVETVEPSFVVFWKYIIHWMFVAVIKEAQRNSKGFPKGALQCCEYI
HKD*

C

AF293 ‘*bafB*’ Chr. 3 Region Translated

MVWYRAILICMLWWLMGYNSTNKGK*SEGKRAPMINKVPTFEEIIITTSAKYVNSEKIIE
HTVIKTKQINN*GDTSISNNDSNSTIETRYSGLSSVSYSD*SKVVKDTNALEEPELFAIY
SSYINASTSK*MLRLYYELPVSLDNLKITGLESRIPESNNDSIKACFCYWGEKFWLYFPY
SYAKARMVLIGVY
