# Supplementary material for: A Heterogeneously Expressed Gene Family Modulates the Biofilm Architecture and Hypoxic Growth of Aspergillus fumigatus
Source: mBio. 2021 Feb 16;12(1):e03579-20. doi: 10.1128/mBio.03579-20 (PMC8545126; doi:10.1128/mBio.03579-20)

A

```
Predicted       MAWYRALLPCIPLWRKVLGRNSTDEDGRSEDDLTSLSDKMPTFEETTTSAKYINGEKIME    60
An08g12010      MAWYSALLPCMLWWRNLLWRNSTNRYRQSTDDLTSLTDKIPNLGERLPNLLSFV------    54
                **** *****:  **::* ****:.  :* ******:**:*.: *   .   :

Predicted       HTVVETKHIDERGDTSVSNGDSNSTAVTRHSGLSSVSLSDQSTIVEDANALEEPELFAVH    120
An08g12010      ----------NSIPDQST-----------------------TAGKINHKALRDPELFAIR    81
                          :.  * *.                       ::   : :**.:*****::

Predicted       SPYVDDSTGEQMVRLYYELPVSLDDLEIIGLESRIPESDDDSIEARFRYRGEDFWLPVRY    180
An08g12010      SSCIDKSASKWMVSLYYEPPPSLDDLEIKNFGSRIPESEDDPIEAIFHYEGENIWVSVPY    141
                *  :*.*:.: ** **** * ******* .: ******:** *** *:*.**::*: * *

Predicted       SYAKARMVLTGVC   193
An08g12010      LYARTRSLSSGLF   154
                 **::* : :*:
```

B

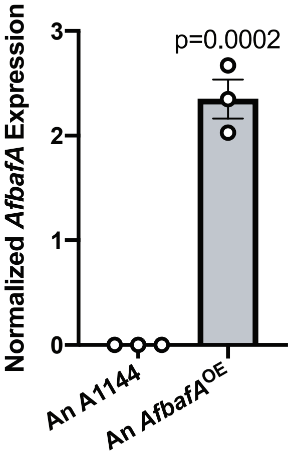

Supplement: FIG S7 [file mbio.03579-20-sf007.pdf]
