# Supplementary material for: A Heterogeneously Expressed Gene Family Modulates the Biofilm Architecture and Hypoxic Growth of Aspergillus fumigatus
Source: mBio. 2021 Feb 16;12(1):e03579-20. doi: 10.1128/mBio.03579-20 (PMC8545126; doi:10.1128/mBio.03579-20)
Supplement: TABLE S2 [file mbio.03579-20-st002.pdf]

Strain ID/Purpose	Primers and Template DNA information
aΔ*cgnA*^{EVOL}; *cgnA*^{RECON} (*cgnA*^{RECON})	**Template: AF293 genomic DNA**
	5'-AAAAggcgcgccGAACGCTGTTTGTGACGTACTCTGTTAT-3'
	5' - AAAAttaattAAGCCAGTCAGTCAGGGAAAAGCAAag - 3'
Δ*cgnA*^{EVOL}; *cgnA*^{OE}	**Template: AF293 genomic DNA**
	5' - AAAAAAAAGGCGCGCCATGGTATTGATCATCGAGATCATCAAGATTG -3'
	5' - AAAAAAAAGCGGCCGCTAGCATCCAAGCGGCCTCTCCTCT-3'
Δ*hrmA*^{EVOL}; *bafB*^{OE}; ΔcgnA^{EVOL}; bafB^{OE}; AF293 bafB^{OE}; CEA10 bafB^{OE}	**Template: CEA10 genomic DNA**
	5' - AAAAAAAAGGCGCGCCatggtgtggtatagggccatactcg - 3'
	5' - AAAAAAAAGCGGCCGCcaagtcagaccattcagtatacaccc - 3'
AF293 *bafA*^{OE}; CEA10 bafA^{OE}; An AfbafA^{OE}	**Template: AF293 genomic DNA**
	5' - AAAAGGCGCGCCatggcttggtatagagccttactc - 3'
	5' - AAAAGCGGCCGCtcagcatacacccgttaaaaccattc - 3'
AF293 *bafC*^{OE}; CEA10 bafC^{OE}	**Template: CEA10 genomic DNA**
	5'- AAAAGGCGCGCCATGGCTTGGTATGAAGTCTTCGAGC- 3'
	5- AAAAGCGGCCGCTCATTCAGTGGGACCGCCTGA - 3'
Δ*cgnA*^{EVOL}; *bafB*$^{OE\text{-}GFP}$	**Template: GFP plasmid pSD66.1**
	5' - ggagctggtgcaggcgctggagcc - 3'
	5' - gagatagagaattgtgtgggatgagagg - 3'
	**Template: CEA10 genomic DNA**
	5' - attttcctgctctccccaccag - 3'
	5' -GGCTCCAGCGCCTGCACCAGCTCCgtatacacccattaaaaccattcgagc - 3'
	**Second round overlap PCR primers:**
	5' - AAAAAAAAGGCGCGCCatggtgtggtatagggccatactcg - 3'
	5' - AAAAGCGGCCGCagagcattgtttgaggcgaccggt - 3'
real-time qPCR primers: *bafA*	5' - GCATACCGTTGTGGAGA - 3'
	5' - GCCCAGAATGTCTGGTTA - 3'
real-time qPCR primers: *bafB*	5' GGAAGAACCTGAACTCTTTG - 3'
	5' AGATCATCGAGGCTTACTG - 3'
real-time qPCR primers: *bafC*	5' CACGGTCGACTTCTTCTA - 3'
	5' -GGAAGAGAGCCTCAATCA - 3'
real-time qPCR primers: *actA*	5' - TCACTGCCCTTGCTCCCTCGTC - 3'
	5' - GCACTTGCGGTGAACGATCGAA - 3'
real-time qPCR primers:t*ub2*	5 - ATAATGTTCAGACCGCCCTCTGCT -3'
	5' - GACGGATGTGGAATTGCCCACAAA - 3'
real-time qPCR primers:*hrmA*	5' - TCATCGACACGACAGATT - 3'
	5' - ATTCACCGCTGCATAGA - 3'
